# Fatal head and neck injuries in military underbody blast casualties

**DOI:** 10.1136/jramc-2018-000942

**Published:** 2018-04-21

**Authors:** Sarah K Stewart, A P Pearce, Jon C Clasper

**Affiliations:** 1 Academic Department of Military Surgery and Trauma, Royal Centre for Defence Medicine, Birmingham, UK; 2 Centre for Blast Injury Studies, Department of Bioengineering, Imperial College London, London, UK; 3 Department of Trauma and Orthopaedics, Frimley Park Hospital, Frimley, UK

**Keywords:** blast, brain injury, underbody blast, survivability

## Abstract

**Introduction:**

Death as a consequence of underbody blast (UBB) can most commonly be attributed to central nervous system injury. UBB may be considered a form of tertiary blast injury but is at a higher rate and somewhat more predictable than injury caused by more classical forms of tertiary injury. Recent studies have focused on the transmission of axial load through the cervical spine with clinically relevant injury caused by resultant compression and flexion. This paper seeks to clarify the pattern of head and neck injuries in fatal UBB incidents using a pragmatic anatomical classification.

**Methods:**

This retrospective study investigated fatal UBB incidents in UK triservice members during recent operations in Afghanistan and Iraq. Head and neck injuries were classified by anatomical site into: skull vault fractures, parenchymal brain injuries, base of skull fractures, brain stem injuries and cervical spine fractures. Incidence of all injuries and of each injury type in isolation was compared.

**Results:**

129 fatalities as a consequence of UBB were identified of whom 94 sustained head or neck injuries. 87 casualties had injuries amenable to analysis. Parenchymal brain injuries (75%) occurred most commonly followed by skull vault (55%) and base of skull fractures (32%). Cervical spine fractures occurred in only 18% of casualties. 62% of casualties had multiple sites of injury with only one casualty sustaining an isolated cervical spine fracture.

**Conclusion:**

Improvement of UBB survivability requires the understanding of fatal injury mechanisms. Although previous biomechanical studies have concentrated on the effect of axial load transmission and resultant injury to the cervical spine, our work demonstrates that cervical spine injuries are of limited clinical relevance for UBB survivability and that research should focus on severe brain injury secondary to direct head impact.

**Key messages**

- Severe head injuries are a leading cause of death from underbody blast (UBB).
- Cervical spine injury, as a result of high rate flexion and extension has previously been described as an important injury mechanism but our results show it not to be clinically relevant.
- Future mitigative strategies will require an understanding of the relationship between axial load and head impact.
- Protection of the head or modification of vehicles could improve survivability from UBB.

## Introduction

The improvised explosive device (IED) evolved to become the primary cause of fatality in recent UK operational campaigns.^1–3^ Based on the environment at the time of the incident, the injuries can been classified into two main types. ‘Mounted’ IED casualties pertain to injuries sustained to personnel in vehicles, from buried devices, whereas ‘dismounted’ IED casualties relate to personnel sustaining injuries while on foot.^4^ The predominant injury mechanisms in dismounted injury are from either the blast wave (primary blast) or energised fragments (secondary blast).^5^ In contrast, the primary loading mechanism of UBB is high rate vertical acceleration as a consequence of energy transmission through the hull of a vehicle.^6^ This underbody loading may be considered a form of tertiary blast injury (blunt impact) but is at a higher rate and somewhat more predictable than injury caused by more classical forms of tertiary injury such as building collapse.

A retrospective analysis by Singleton *et al* described differences in blast mortality between mounted and dismounted casualties in a UK cohort. In the mounted group, central nervous system (CNS) injuries were the most common cause of death (50%).^6^ However, this data encompassed both head and proximal neck injuries and no further mechanistic detail was given.

It has been postulated through biomechanical studies that mounted head and neck injuries from IED blasts are sustained as a result of the vertical dynamic load propagated through the neck, causing injury through compression, flexion and tension forces.^6^

Existing data supports axial loading as the primary mechanism for other injury types. Pelvic injury patterns in IED blasts are different between dismounted and mounted casualties with the latter associated with injuries to the lower spine, suggestive of direct loading through the seat.^7^ In addition, work by Spurrier *et al* has shown that a biomechanistic pattern of direct loading in mounted IED blast injuries is replicated in the thoracolumbar spine.^8^ His retrospective case series of UK military personnel who sustained in-vehicle blast injuries demonstrated that wedge compression injuries of the thoracolumbar spine predominated. This study also described a much smaller number of cervical spine fractures, but overall axial loading through the neck in concordance with injury patterns identified in the pelvis and thoracolumbar spine for mounted IED blast injuries was the most common mechanism of cervical spine fracture.^8^

However, what is not clear from the literature is the specific mechanism of injury that is associated with fatal CNS injury, and whether isolated axial load, via the cervical spine is sufficient to cause death, or whether associated head impact, or bending at the upper cervical spine are also required. This is particularly relevant as Singleton noted that the fatal CNS injuries were beyond current medical management, and therefore, injury prevention offers most hope of improving survival.^9^

The aim of this study was to evaluate, in greater detail, the CNS injuries sustained by mounted UK personnel killed in the recent conflicts, focussing specifically on those associated with a cervical spine injury. Through identification of injury aetiology, biomechanistic hypotheses may be determined, with scope for the development of strategies for future mitigation and prevention of this injury subset.

## Methods

The UK Joint Theatre Trauma Registry (JTTR) is a trauma database that contains prospective data for all UK military personnel injured or killed while on deployed operations. With the permission of the medical director (Defence Medical Services) and the Royal Centre for Defence Medicine, a search was carried out on the JTTR for injured tri-service personnel from 2003 to 2014 during Iraq and Afghanistan operations. Vehicle occupant non-survivors of IED explosions were identified. Explosive mechanisms other than underbody IED or mine (such as rockets or grenades) were excluded in an effort to control for axial loading only.

Information on cause of death was obtained through collection of data from postmortem reports. This has been described in detail elsewhere.^10^ In brief, postmortems were performed following repatriation to the UK and the pathologists findings were coded using the Abbreviated Injury Scale (AIS). The AIS is a scoring system that identifies nine body regions (head, face, neck, thorax, abdomen, spine, upper extremities, lower extremities and external) and uses an anatomic ordinal scale to score trauma severity, from one (minor injury) to six (maximum injury, currently unsurvivable). The scale was originally developed by the Association for the Advancement of Automotive Medicine and has been regularly updated with recent updates reflecting the need for improved description of military relevant injuries.^11–13^ Although useful for ranking injury severity, these scores are based on consensus opinion with reference to outcome. The injury scale is neither linear nor well correlated with mechanical input and so of relatively little use for the purposes of forensic biomechanical analysis. Instead, by examining postmortem data, a biomechanically based pragmatic approach has been used to delineate head and neck injuries in a complex trauma patient, which are likely to have contributed to death based on the score of the neck and neck regions. AIS has utility for the exclusion of minor injuries and any cases with no injuries graded AIS two or above were excluded as this implies non-critical injury.

Head and neck injuries were classified in to five regions according to anatomical site of injury: skull vault fractures, parenchymal brain injury, base of skull fractures, brain stem injuries and cervical spine fractures.

## Results

From 2003 to 2014, there were 129 recorded fatalities on the JTTR from mounted IED blasts. Mean age of the group was 25.9 years (SD 5.6). Median NISS and ISS was 75 (range of 30–75 for both). Ninety-four out of 129 (74.4%) had at least one head or cervical spine injury identified. Of the 94, the median age was 25, and 92 were male (97.8%).

A further seven casualties were excluded as they sustained such devastating injuries, with tissue loss of the head and neck that it was impossible to determine the mechanistic cause. Of the remaining 87, the most common site of injury was parenchymal brain injury (Table 1). All cervical spine injuries were fractures, there were no significant soft tissue only injuries to the cervical spine, although six of the 35 excluded casualties (17.1%) had sustained significant soft tissue injuries to the anterior structures of the neck.

**Table 1:** Incidence of injuries by site of injury

Site of injury	Number (%)	Comment
Skull vault injury	48 (55.2)	Twenty-five had associated basal skull fracture
Parenchymal brain injury	65 (74.7)	
Basal skull fracture	40 (46.0)	Eleven had associated brain stem injury
Brain stem injury	28 (32.2)	Eighteen had associated vault or basal skull fracture
Cervical spine fracture	16 (18.4)	See text for further details

From Table 1, it can be calculated that the 87 casualties sustain a total of 197 injuries, with an average of 2.26 regions injured per casualty, suggesting multiple injuries were most common. Only 25 of the 87 (28.7%) casualties had a single region injury (Table 2).

**Table 2:** Frequency of isolated injuries by site of injury

Site of injury	Number (%)
Multiple sites	62 (71.3)
Isolated skull vault injury	4 (4.6)
Isolated parenchymal brain injury	12 (13.8)
Isolated basal skull fracture	7 (8.0)
Isolated brain stem injury	1 (1.1)
Isolated cervical spine fracture	1 (1.1)
Total	87

Of the 16 cervical spine fractures, six (37.5%) were minor, stable fractures unlikely to threaten life. Of the 10 (62.5%) significant cervical spine fractures, eight were in casualties with very severe multisystem injuries, who would have died even if the cervical spine was intact. In only two patients (2/87=2.3%) did the cervical spine injury appear to be the most severe injury. One patient died with a displaced C1/2 fracture associated with subtotal cord transection, but also had a severe brain stem injury; possibly as a result of an extension/axial loading mechanism. The other patient also had a C2 injury, associated with severe cord contusion and a basal skull fracture; this was the only casualty where isolated axial loading appeared to be the mechanism of injury to the cervical spine.

Only two of the 16 cervical spine fractures (12.5%) were associated with a brain stem injury, providing weak support for a mechanistic link.

## Discussion

Although the majority of wartime trauma involves the limbs, it is injuries to the head, torso and junctional haemorrhage from the neck, axilla or groin that are the most life threatening.^3^ Mitigating strategies for prevention of these devastating injuries is therefore indicated. Mounted blast casualties represent a major cause of morbidity and mortality among service personnel, as a result of the high energy blast wave that is required to propagate through the vehicle to disrupt both it and its occupants. Recent work has shown that the aetiology of mounted blast fatalities is distinct from dismounted blast fatalities, with death arising primarily as a result of head injury in the mounted population compared with junctional and extremity haemorrhage in the dismounted population.^9^

Although cervical spine injuries have been reported from recent conflicts,^14–17^ this is the first report to specifically focus on the role of the cervical spine in relation to mortality following mounted blast; this is part of our group’s focus on ‘Future Unexpected Survivors’.^9^

Three hypotheses can be suggested regarding the mechanism of cervical spine injuries in mounted IED blast fatalities. First, injuries can be caused by direct trauma to the cranium as a result of the head striking the vehicle roof or other object. In this instance, cervical spine injuries would be caused by the transmission of force from above, prevention/mitigation would be preventing this impact or optimising helmet design and head rather than cervical spine protection is probably more important. Based on our results, this would appear to be the most common mechanism of fatal head injury in mounted casualties.

Second the force may be transmitted from below and from the cervical spine to the base of the skull, and the main aim of this work was to analyse this possible mechanism. This vertical pattern of loading has been postulated in other studies looking at traumatic cervical spine injuries^14 15^ and would be consistent with work where axial loading is responsible for the injuries seen in the blasted pelvis and spinal column. We were only able to identify one fatality where this mechanism was likely.

A third hypothesis is that displacement of the neck through a compression/bending mechanism gives rise to brainstem injuries and high cervical spine injuries. This is in keeping with Spurrier *et al*’s work examining spinal injuries patterns in mounted blast casualties.^8^ Spurrier *et al* identified a high incidence of wedge compression injuries in the thoracolumbar spine, postulating that they arose through forward flexion of the spinal column as a result of an axial load being transmitted anterior to the spine. Again, we were only able to identify one fatality where this mechanism may have caused death.

Although we could find no convincing evidence to support the cervical spine as a specific target for prevention or mitigation strategies, significant injuries did occur, and were consistent with death due to axial loading to the upper cervical spine/base of skull. In two incidents, involving two different types of vehicles, there was a fatality with evidence of cervical spine displacement into the base of skull, consistent with previous theories of vertical dynamic loading.^6^ However, both of these casualties sustained significant vertical loading injuries to other body regions, incompatible with survival, and so prevention/mitigation should be directed to whole body loading rather than focusing on the upper cervical spine.

In addition, the mounted blast casualty is exposed to a number of different injury mechanisms; in one particular incident, we found evidence of death from injuries normally seen in dismounted fatalities, with multiple traumatic amputations and pelvic disruption, possibly from flail. However, a second fatality from the same incident had significant axial loading injuries, with head impact and no traumatic amputation, but both casualties had similar spine injuries. The complexity and relative unpredictability can be further illustrated, as a casualty sitting close to both fatalities escaped relatively uninjured. Dismounted blast injuries will be more likely to occur with open top vehicles, or if vehicle breech occurs, and so the range of vehicles, an individual’s position within the vehicle, as well as the explosive size also makes specific mitigation strategies more complex.

The main weakness of this study is the retrospective nature. However, the JTTR consists of specific, prospectively collected data, from multiple sources. In particular the postmortem is attended by specifically trained trauma nurse coordinators, who add free text as well as coding the injuries. In addition, it will be revised as and when further data becomes available, from radiology reports, particularly postmortem CT analysis. It should also be emphasised that studies like this are part of the raison d’etre for the JTTR, and so its structure is related to clinical governance, rather than just being a generic database. The limitations of AIS and other trauma scoring systems are recognised by the UK Defence Medical Services.^18^ The importance of these tools arises from the highlighting of both trends and outliers for further analysis and research.

The fact that we focused on fatalities, most of whom died at the time, or recently soon after the incident negates the effect of any medical intervention. As CT analysis was undertaken soon after injury, even in fatalities, the observed fracture patterns allow accurate analysis of injury mechanism. It must be acknowledged that the soft tissue injuries to the brain parenchyma and brain stem may be an underestimate of the true incidence, as discrete, but significant injuries, and even diffuse axonal injury may be present, but not evident if death was instant. However, this does not alter the message that the cervical spine is not a significant direct cause of death after mounted blast and, in isolation, should not be a major focus of prevention/mitigation strategies.

## Conclusion

We have demonstrated that fatal head injuries from in-vehicle blasts do not appear to be mechanistically related to cervical spine injury. Death appears to be related to blunt trauma to the cranium, but with significant axial loading resulting in multiple injuries. Further research is needed to develop strategies to mitigate against these injuries, through mitigation of axial loading, and further protection to the head or prevention of impact.
